# City Hospital of Chicago

**Published:** 1859-09

**Authors:** 


City Hospital of Chicago.—This new hospital has been completed, and the staff of surgeons and physicians consists of the following gentlemen:—

*Surgeons.*—Drs. Brainard, Amerman, and Schloetzler.

*Physicians.*—Drs. Miller, Blake, and Ross.

